# Preoperative tumor marking with indocyanine green prior of robotic colorectal resections

**DOI:** 10.3389/fsurg.2022.1087889

**Published:** 2022-12-22

**Authors:** Michael K. Konstantinidis, Argyrios Ioannidis, Pantelis Vasiliou, Nikolaos Arkadopoulos, Ioannis S. Papanikolaou, Manish Chand, Tom Pampiglione, Dimitrios Karagiannis, Konstantinos Konstantinidis

**Affiliations:** ^1^ Department of General, Laparoscopic, Oncologic and Robotic Surgery, Athens Medical Center, Athens, Greece; ^2^ Fourth Department of Surgery, Attikon University Hospital, National and Kapodistrian University of Athens School of Medicine, Athens, Greece; ^3^ Hepatogastroenterology Unit, Second Department of Internal Medicine – Propaedeutic, Medical School, National and Kapodistrian University of Athens, Attikon University General Hospital, Athens, Greece; ^4^ UCL Division of Surgery and Interventional Sciences, WEISS Centre, University College London, London, United Kingdom; ^5^ Department of Gastroenterology and Hepatology, Athens Medical Center, Athens, Greece

**Keywords:** colorectal surgery, indocyanine green, near-infrared fluorescence imaging system, fluorescent guided surgery, robotic surgery

## Abstract

This prospective case-series study aimed to assess the usefulness of preoperative colonoscopic marking of colorectal tumors using Indocyanine Green (ICG) fluorescence in patients that underwent robotic surgical colorectal resections. Consecutive patients that were eligible for colorectal resection with intent to cure in a single hospital (Athens Medical Center), from February 2022 to June 2022, were included. ICG solution was injected into the submucosal layer at 2 opposite sites (180 degrees apart) distal to the tumor, without submucosal elevation. Identification of the tumor marking was then performed after switching to near-infrared (NIR) fluorescence mode. During the robotic procedure, qualitative evaluation of fluorescence was performed by the surgical team (primary surgeon, first assistant, second assistant, research fellow). All 10 patients underwent robotic surgical approach and operations included right-sided colectomy (*n* = 1), left-sided colectomy (*n* = 6) and low anterior resection (*n* = 3). Visualisation of this dye with near-infrared light was very clear with bright intensity in all patients when the marking was performed one day prior of surgery. Preoperative tumor marking with ICG was identified intraoperatively in all cases and the techinque was easily reproducible.

## Introduction

Minimally invasive robotic surgery for patients with colorectal cancer is increasingly being preferred over conventional surgery due to its comparable survival and recurrence rates along with improved visualisation and dexterity with which complex dissection can be carried out (1, 2). However, intraoperative detection of neoplasms has been challenging due to lack of tactile sensations (3). A variety of techniques have been used to detect colorectal tumors including barium enema, colonoscopic metallic clipping, computed tomography colonoscopy, intraoperative colonoscopy as well as preoperative colonoscopy. Localization and marking of tumors preoperatively by endoscopists is of crucial significance and offers surgeons anatomical guidance especially in complex cases. Detection of colorectal lesions with endoscopic tattooing has been reported since 1975 (4).

India ink has been traditionally used to mark tumors *via* colonoscopic tattooing due to its effectiveness and accuracy in detection of small lesions. However, reports of side effects such as inflammation, local peritonitis, abscesses and adhesions have been recognized. Furthermore, a major disadvantage that has been observed is spillage of India Ink out of serosa. This agent cannot be eliminated and stays permanently in the tissues, potentially altering the surgical anatomical plane (5, 6).

Indocyanine Green (ICG) has been described as a potential agent for preoperative colonoscopic marking in animal models since 1989 (5–7). This fluorophore has several applications in colorectal surgery, such as assessment of bowel perfusion (8). ICG fluorescence imaging as tumor site marking in near-infrared (NIR) fluorescence has been reported as practical due to its lower surgical view interference by being less visible in white light (9–12). It is also safe with fewer and more tolerable side effects being observed in contrast to India Ink, although more comparative studies are needed. However, there are some facts that have not yet been predetermined regarding preoperative ICG tumor marking. The timing between local ICG injection and surgery for maximal visualization has not yet been defined. Moreover, several methods have been reported regarding the dosage and the technique used by endoscopists in order to inject this agent efficiently. In this study, we aimed to evaluate the usefulness of preoperative colonoscopic marking of colorectal tumors using ICG in robotic colorectal resections for cancer in a single hospital carried out by a single surgical team (Athens Medical Center).

## Methods

### Study design

From February 2022 to June 2022, consecutive patients from Athens Medical Center eligible for colorectal resection were enrolled in this prospective case-series study. Included patients were required to be at least 18 years old with histopathologically confirmed colorectal adenocarcinoma. They underwent preoperative colonoscopic tumor marking with ICG less than 24 h prior of the operation. Patient demographics were collected and included gender, age, body mass index, American Society of Anesthesiologist class and preoperative stage (Table 1). Patients who had previously experienced an adverse reaction to ICG and/or iodine, cases with obstructed colon requiring emergent operation, metastatic disease as well as pregnant women were excluded.

**Table 1:** Patient characteristics.

*N*	10
Sex ratio (Males:Females)	6:4
Age (years)^a^	67 (56–80)
Body Mass Index (kg/m²)^a^	25.3 (20.1–31.7)
Smoking: Yes, No	7, 3
ASA Classification	I:3
	II: 5
	III: 2
	IV: 0
Preoperative tumour staging	
T1	1 (10)
T2	2 (20)
T3	7 (70)
T4	0 (0)

ASA, American Society of Anesthesiologists.

Values in parentheses are percentages.

^a^ Values are median (range).

### Ethical statements

This study was approved by Athens Medical Center Institutional Review Board. Informed consent was obtained from all participants after comprehension and agreement with the study's protocol.

### Study procedure

Under sterile conditions, solution of ICG was prepared by dissolving 25 mg of ICG (Verdye™) in 10 ml of sterile water (2.5 mg/ml solution). Using a 25-gauge needle, 0,1 ml of ICG solution was injected into the submucosal layer at 2 opposite sites (180 degrees apart) distal to the tumor. We did not use submucosal elevation with normal saline. The minimum volume that could be technically administered without difficulties was selected as the local injection dosage.

**Figure 1:**
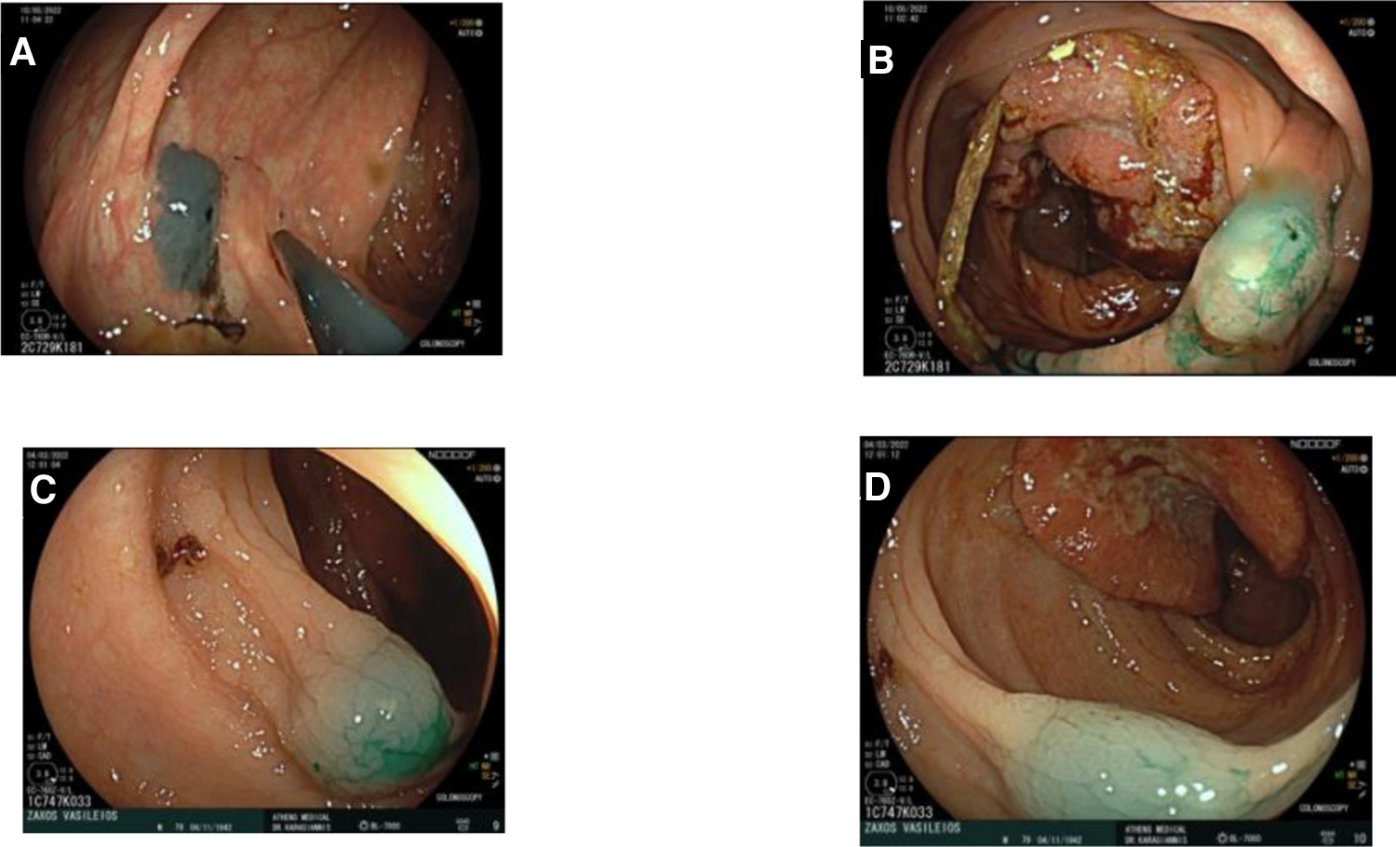
Endoscopic tattooing using indocyanine green in two patients. (**A**) Patient 1: Injection distally of tumor, one side. (**B**) Patient 1: Injection distally of tumor, other side. (**C**) Patient 2: Injection distally of tumor. (**D**) Patient 2: Injection distally of tumor.

**Figure 2:**
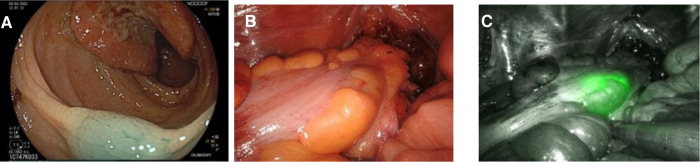
Three images of the same patient marked with indocyanine green. (**A**) Endoscopic view of marked tissue with Indocyanine Green*.* (**B**) Intraoperative view of rectosigmoid junction during robotic surgery under white light. (**C**) Intraoperative view of rectosigmoid junction during robotic surgery under Near-Infrared visualization mode.

During the preoperative marking approach, patients were positioned in left lateral position and were marked by an expert gastroenterologist (D.K.). During surgery, entry to the peritoneum was approached using Hasson technique, with a 8-mm trocar to house the endoscope. Pneumoperitoneum was induced and maintained at 12 mm Hg. After the placement of trocars for working and assist ports, the robotic 30° camera was inserted and the peritoneal cavity was explored using standard, high-definition, white light imaging. Identification of the tumor marking was then approached after switching to NIR fluorescence mode (Figures 1, 2).

### Outcomes

Qualitative evaluation of fluorescence was performed by the surgical team (main surgeon, main assistant, second assistant, research fellow). Intensity of fluorescence of the marked colonic site was subjectively evaluated due to the lack of any objective technique for quantification.

Patient information, pre-operative radiological staging, type of operation, histological lymph node yields, and histological staging were also documented.

## Results

During the study period, February 2022 to June 2022, ICG-enhanced fluorescence was used as tumor marking in 10 patients (4 females). Patient and tumor characteristics are presented in Table 1. All patients underwent robotic surgical approach and operations were categorised as right colectomy (*n* = 1), left-sided colectomy (*n* = 6) and low anterior resection (*n* = 3) (Table 2).

**Table 2:** Operation type.

Right-sided Colectomy	1 (10)
Left-sided Colectomy	6 (60)
Low Anterior Resection	3 (30)

Values in parentheses are percentages.

All patients were marked solely with ICG. In all patients visualization of fluorescent tissues was adequate with bright intensity and clear separation of marked and non-marked sites (Table 3). Median time for tumor identification under NIR fluorescent mode was 2 min. All tumors were entirely removed, with negative resection margins. There were no complications attributed to the ICG as well as neither intraoperative nor postoperative adverse events or conversion to open surgery (Tables 4, 5).

**Table 3:** Intraoperative ICG details.

- Identifiable Tumour: Yes	10 (100)
- Tumor marking intensity: Very Bright	10 (100)
- Timing for Tumour Identification (min)^a^	2 (1.5–5)
- Spillage of ICG: No (%)	10 (100)
- Adverse Events: No	10 (100)

ICG, indocyanine green.

Values in parentheses are percentages.

^a^ Values are median (range).

**Table 4:** Perioperative clinical results.

Operation time (min)^a^	180 (120–350)
Estimated blood loss (ml)^a^	50 (30–100)
Complications: Clavien Dindo Classification	I: 3
	II: 1
	III: 0
	IV: 0
Hospital Stay (days)^a^	5 (4–8)

Values in parentheses are percentages.

^a^ Values are median (range).

**Table 5:** Summary of results.

Age	Sex	Tumour Location	Operation	Preoperative Tumor Marking with ICG	Post operative Staging	Dissected Lymph Nodes
67	M	Sigmoid	LSC	Yes	T2N0M0 R0	18
71	F	Sigmoid	LSC	Yes	T2N1M0 R0	16
68	F	Sigmoid	LSC	Yes	T2N0M0 R0	20
72	M	Sigmoid	LSC	Yes	T2N1M0 R0	16
77	M	Rectum	LAR	Yes	T1N0M0 R0	17
69	F	Sigmoid	LSC	Yes	T3N1M0 R0	18
68	M	Cecum	RSC	Yes	T2N0M0 R0	19
73	M	Sigmoid	LSC	Yes	T3N1M0 R0	15
74	F	Rectum	LAR	Yes	T2N0M0 R0	20
69	M	Rectum	LAR	Yes	T2N0M0 R0	15

LSC, left-sided colectomy; LAR, low anterior resection; RSC, right-sided colectomy.

## Discussion

During minimally invasive surgery, identifying lesions by palpation is impossible due to the fact that tactile feedback can not be achieved. The surgeon must rely on visual evaluation in combination with preoperative imaging in order to be guided for proper surgical resection. A variety of techniques have been described for detection of colorectal tumors including preoperative barium enema, CT scans, CT colonography, proctoscopy with stitch as well as colonoscopy with metallic clipping or tattoo (13–15). Barium enemas are ineffective in visualizing small tumours (15, 16). Metallic clip use is insecure due to occasional low visibility as well as migration to other tissues (14). Narihiro et al. observed the safety and effectiveness of near-infrared fluorescent clips and reported a detection rate 94.1% without adverse effects related to clip marking (17). Intraoperative colonoscopy can be used to identify GI lesions, however this extends the overall duration of the operation and can generate intestinal distention, which might limit the surgeon's surgical field (14, 18). Preoperative colonoscopy with simultaneous tumor marking using a dye may be necessary to precisely determine the level of the tumor and perform the appropriate excision. An alternative approach has also been described by using patients autologous blood instead of a dye (19–21). Kim et al. used 6–12 ml of autologous blood for endoscopic tattooing and reported a visualization rate of 92.2% with three patients (5.9%) experiencing endoscopic adverse effects related to the technique (19).

Preoperative colonoscopy with simultaneous injection of a dye in the intestinal wall is currently the most efficient and widely used way for identifying colorectal lesions. Multiple dyes that have been tested in animals including India ink, ICG, methylene blue, indigo carmine, toluidine blue, and isosulfan blue. However only India ink and ICG were detectable up to 48 h after marking (7, 22, 23). India ink has traditionally been used to mark tumors *via* colonoscopic tattooing due to its effectiveness and accuracy in detection of small lesions. Currently, it is the most commonly used agent for tattooing. However, reports of side effects such as inflammation, local peritonitis, abscesses and adhesions have been recognized. Furthermore, a major disadvantage that has been observed is in the case of spillage of India Ink out of serosa (5, 23, 24). This agent cannot be eliminated and stays permanently in the tissues confusing surgeons in the observation of the correct anatomical plane. The use of ICG gives an alternate means of correctly detecting and identifying the tumor to provide appropriate resection margins without the issues outlined above (5, 24, 25).

Several experimental research comparing ICG and India ink for colonic tattooing in animals found that India ink outperforms ICG due to its higher visibility and longer duration. A longer period is not usually required for surgical resection. In human cases, endoscopic tattooing with ICG was evident 36 h after injection in 12 individuals and resulted in relatively minor complications (5, 7, 22, 23).

In the current literature, different approaches have been described regarding the day of the tumor marking prior to the surgery as well as the dosage and the concentration of the solution. According to Miyoshi et al., after injection of 1 ml of 1.25% ICG, it could be clearly detected in all 29 patients who underwent surgery within 8 days, whereas it was visible in only 2 of 10 patients who were operated after 8 days (24). Similar results were reported by Satoyoshi et al. who injected 0.1 ml of 0.5% ICG and evaluated a total of 100% visibility when the marking was performed within 6 days preoperatively as opposed to 60% and 0% when it was done between 7 and 9 and over 10 days, respectively (25). Watanabe et al. used 0.5 ml of 0.25% ICG which was sufficiently visible when the procedure was performed up to 7 days prior (11). Alternatively, Kim et al. injected 0.5–1 ml of 1.25% ICG a day prior of surgery and compared the measured outcomes to a group of non-tattooed patients, reporting a shorter operation time, hospital stay and postoperative oral ingestion period in favour of the tattooed group (18).

The purpose of this study was to evaluate the use of ICG as a preoperative tumor marking dye and describe the technique that we have been using in order to maximise the potential benefits of this procedure. As opposed to the currently standard method of tumor marking which is India Ink injection following submucosal elevation with saline test injection, we injected ICG directly to the submucosa without saline test injection. ICG may be managed more effectively than India Ink and injected straight into the submucosa without the need for a saline test injection due to the fact that it does not contain particles (18). The benefit of the direct injection technique is that there is no need of changing syringes. One of our findings is that visualisation of this dye with near-infrared light is very clear when the procedure was done one day prior of the surgery. Separation of marked and unmarked tissues was easily evaluated during laparoscopy and guided the surgeon for rapid recognition of anatomical landmarks. Injection of the minimum dosage (0.1 ml) of diluted solution (2.5 mg/ml) was performed in order to minimise the risk of spillage to surrounding tissues. This study adds to the little evidence available on the usefulness of ICG in this scenario.

There were no adverse events, which adds to the evidence that ICG is safe in minimally invasive colorectal surgery. This study provides preliminary evidence of the safety, feasibility and reproducibility of employing ICG to intraoperatively localize colorectal tumours.

However, this study has some limitations. It is an observational study which lacks comparison between techniques. Additionally, there is no specific method to accurately quantify the intensity of the signal and the interpretation of the images is under the surgical team's discretion. For that reason, subjective evaluation of fluorescence is still regarded as biased. An other limitation is the small sample size which reduces the power of the study. A randomised prospective comparative study, using as control group patients who have been preoperatively marked with India Ink, would be necessary in order to demonstrate whether the benefits of this technique are significant for tumor localisation and possible adverse events. In addition, more studies are required to also verify the optimal dosage of the fluorophores, as shown recently by an intercontinental experts Delphi consensus study (26).

## Conclusion

Visualisation of preoperatively marked tumors with ICG offers a great choice that could potentially be considered by surgeons before colorectal procedures. The intraoperative view of previously marked tissues under near-infrared light can be very clear with bright intensity. Separation of marked and unmarked tissues can be easily evaluated during laparoscopy and guide the surgeon throughout the operation.

## Data Availability

The original contributions presented in the study are included in the article/Supplementary Material, further inquiries can be directed to the corresponding author/s.
